# A Novel Canine Mammary Cancer Cell Line: Preliminary Identification and Utilization for Drug Screening Studies

**DOI:** 10.3389/fvets.2021.665906

**Published:** 2021-05-27

**Authors:** Rifei Li, Haoxian Wu, Yue Sun, Jingru Zhu, Jun Tang, Yu Kuang, Gebin Li

**Affiliations:** College of Veterinary Medicine, China Agricultural University, Beijing, China

**Keywords:** canine mammary tumor, cell line, characteristic, drug, cell cycle arrest

## Abstract

Canine malignant mammary tumor is a dangerously fatal neoplastic disease with poor survival in female dogs. The aim of this study was to preliminary characterize a novel canine mammary cancer cell line, B-CMT, from canine primary mammary gland tumor, and to utilize it as a cell model for *in vitro* screening of possible therapeutic drugs. The successfully established cell line, B-CMT, was cultured over 50 passages. B-CMT has a fast proliferation rate, and a population doubling time (PDT) of 33.6 h. The B-CMT cell line lacked human epidermal growth factor receptor-2 (HER-2), estrogen receptors (ER) and progesterone receptors (PR) expression by qRT-PCR. Compared with MDCK cells, CDH1 expression of CMT cell line was significantly decreased or even absent, but GATA3 expression dramatically increased, while TGF-β expression was at a similar level. Interestingly, the B-CMT cell line from canine primary tumor also showed positive hypoxia inducible factor-1α (HIF-1α) results in immunofluorescence (IF), western blot, and qRT-PCR analysis. Ten days post inoculation with EGFP-B-CMT (B-CMT cells stably expressing EGFP), the experimental mice developed palpable soft tissue masses which histologically resembled the canine primary tumor, and was approved to be derived from B-CMT cell line through detection of EGFP by immunohistochemical (IHC) analysis. Moreover, we investigated the cytotoxicity of five drugs to B-CMT cells, and the results showed that rapamycin and imatinib significantly inhibited the proliferation of the cells *in vitro* within a certain range of concentration. They also induced cell cycle arrest of B-CMT cells at G1 and G2 phase, respectively. In summary, the results of this report showed that B-CMT cell line might serve as a tool for future studies on tumor microenvironment and drug resistance.

## Introduction

Canine mammary tumor (CMT) is the most common type of tumor occurring primarily in female dogs, rarely in males, leading to high levels of morbidity in China. Mammary tumors often appear as benign and malignant lesions in dogs, with similar incidence rates. Yaritza et al. (1) conducted an epidemiological study of mammary tumors in female dogs diagnosed over a 10-year period (2002–2012). Examination of 1,917 canine mammary biopsies revealed an average annual incidence rate of 16.8%, of which 47.5% were malignant and 47% were benign, respectively. In addition, the average age of dogs having mammary tumors range from 9 to 11 years, with young bitches under 2 rarely having any. Zatloukal et al. (2) reported that the average age of dogs with benign and malignant mammary gland tumors was 8.9 and 10 years, respectively. They also reported that the percentage of malignant tumors increases correlating with age.

The pathological types of CMT are complex, which brings some difficulties to the clinical prognosis of canine mammary gland tumors. Yamagami et al. (3) reported a correlation between malignant canine mammary gland tumors, the TNM staging and histological classification: as tumor size increases, clinical prognosis worsens. For example, the 2-year survival rates of dogs with local lymph node metastasis of mammary tumors were significantly lower than those counterparts without such metastasis. The TNM system describes the amount and spread of cancer in a patient's body, using TNM. Therefore, accurate clinical staging is important for proper assessment diagnosis and prognosis of dogs with mammary tumors.

Treatment of mammary tumors is continuously proven to be significant challenging for veterinarians. Chemotherapy is a classical form of treatment. Using chemotherapeutics to suppress or even kill cancer cells has been reported to prolong the survival of cancer patients (4). Chemotherapy drugs such as adriamycin, carboplatin, and cisplatin have proven to possess certain anti-tumor activity through *in vitro* cytotoxicity testing on 30 different canine mammary cancer cell cultures established from 30 mammary gland tumors excised from dogs (5). Moreover, Karayannopoulou et al. (6) performed adjuvant post-operative chemotherapy in dogs with mammary gland tumor. Sixteen bitches were divided into two groups: one group received 5-fluorouracil in combination with cyclophosphamide for 4 weeks, and the other group received surgical excision alone. Survival analysis demonstrated that the chemotherapy regimen had an active impact on disease-free interval and survival time in chemotherapy treated animals as compared with non-treated. In addition, Simon et al. (7) demonstrated that postoperative adjuvant treatment with doxorubicin and docetaxel of invasive malignant mammary gland tumors in dogs was able to improve long-term local control rate and survival rate in dogs. However, there was no significant difference found in recurrence interval, metastasis time or overall survival rate in their study. Imatinib is a tyrosine kinase inhibitor (TKI) used to clinically treat canine mast cell and gastrointestinal stromal tumor (8). Chen et al. (9) reported that imatinib enhances the antitumor activity of doxorubicin in canine B-cell lymphoma cell. Rapamycin, a specific inhibitor of mTOR, reduces phosphorylation of the mTOR targets, which potentially restrain the growth and proliferation of malignant tumors in dogs (10). Previous studies have shown that rapamycin can effectively inhibit the proliferation of canine osteosarcoma cells *in vitro* (11). The ideal chemotherapy drug should be able to inhibit tumor cell proliferation while limiting cytotoxic effects without harming normal cells. At present, few chemotherapy drugs have completely eradicated tumors and the majority of them have some toxic effects on the body, therefore drug screening is particularly important (12).

Cell lines play a critical and crucial role in tumor research and drug screening. Establishing tumor cell lines *in vitro* can be used to study the biological characteristics of tumor cells and tumor-related molecular pathways. For example, SFRP2 expression in mammary gland tumor cells has been found to be higher than in normal cells and thus may be a potential marker for canine mammary gland tumor cells (13). Improved cell culturing technology has led to the establishment of a number of canine mammary gland tumor cell lines. Studies using four new canine mammary gland cancer cell lines, derived from primary and metastatic lesions, have found that reduced E-cadherin function may be related to the invasion and metastasis potential of canine mammary gland tumor cells (14). In addition, Caceres et al. (15) successfully established a new canine inflammatory mammary cancer (IMC) cell line, designated IPC-366, which is a triple negative cell line characterized to have strong tumorigenicity *in vivo* and high rates of invasiveness and metastases.

Hypoxia-inducible factor HIF-1α, which is induced by hypoxia, plays a crucial role in angiogenesis. Human hematological malignant disease, including leukemia and lymphoma, express high levels of HIF-1α, which is often closely associated with poor disease prognosis (16, 17). However, there are few researches exploring the relationship between cancers and HIF1α in the veterinary field (18). Kambayashi et al. (19) reported that HIF-1α is expressed in the canine lymphoma between cell lines and clinical tissue samples, and that its inhibitor is capable of suppression of cell proliferation both *in vitro* and *in vivo*. Therefore, we focused on the expression of HIF1α in canine cell lines established by us.

Although several canine mammary gland cancer cell lines have been established in the past few years, they are still relatively limited in numbers, and each cell line has its own variant characteristics. Therefore, there is a need to establish new lines with higher diversity representing characteristics of the original mammary tumor which can be useful for researchers. As such, the aim of our study was to establish a new canine mammary cancer cell line (B-CMT) and characterize it, with respect to biological characteristics and tumorigenicity, as well as drug screen sensitivity to provide reference for the treatment of mammary gland cancer.

## Materials and Methods

### Ethics Statement

Eight 4-week-old nude mice were purchased from Beijing Vital River Laboratory Animal Technology Co., Ltd (Beijing, China). All animal procedures and study design were conducted in accordance with the Guide for the Care and Use of Laboratory Animals (Ministry of Science and Technology of China, 2006), and were approved by the animal ethics committee of China Agricultural University.

### Origin and Characteristics of Tumor Specimen

The cell line was derived from a 13-year-old female, sterilized, Pomeranian dog presented to the China Agricultural University Veterinary Teaching Hospital and diagnosed with mammary gland cancer. The dog was originally diagnosed with mammary adenocarcinoma through histopathology in 2015, with nodular masses in the first and second mammary gland areas, and an 2 ×1 cm irregular mass in the middle of the 45th mammary gland area. The tumor was excised surgically without postoperative chemotherapy. The tumor recurred with a mass ~4 cm in diameter, and was diagnosed pathologically as a mixed adenocarcinoma. Tumor samples were immediately processed for histopathological confirmation of CMT and cell culture after the surgical excision. Tumor fragments (2 cm) were placed in Dulbecco's modified eagle medium (DMEM) with 1% penicillin-streptomycin solution.

### Establishment of B-CMT Cell Line

Tumor tissue was fragmented and washed 3 times in phosphate buffer saline (PBS) containing 1% penicillin-streptomycin solution. After washing, the tumor fragments were disrupted and dissociated by resuspension in PBS containing EDTA and 0.25% trypsin, with interrupted agitation for 10 min in a 37°C water bath. Subsequently, the tissue suspension was centrifuged at 1,100 rpm for 5 min, followed by resuspending in DMEM solution supplemented with 10% fetal bovine serum (FBS) and 1% penicillin-streptomycin. The pelleted cell suspension was subsequently centrifuged at 600 rpm for 5 min and supernatant containing tumor cells was collected into a 10 cm wide cell culture dish. The above steps needed to be repeated 3 times to complete the isolation of tumor cells. Tumor cells were maintained in DMEM supplemented with 10% FBS in a 5% carbon dioxide (CO2) humidified atmosphere at 37°C.

Once density confluency reached 90%, cells were then washed three times with PBS and detached using 0.25% trypsin. Trypsinization was terminated using DMEM containing 10% FBS. The resultant cell suspension was centrifuged at 1,000 rpm for 5 min. Fifty percentage of trypsinized cells were reseeded to a new culture dish, whereas the remaining cells were suspended in frozen stock solution containing 90% FBS and 10% dimethylsulfoxide (DMSO), and cryopreserved at −80°C initially then −196°C. The cell line was maintained under continuous subculture conditions for 50 passages, and upon completion of the 50th passage, the established cell line was designated as B-CMT cells.

### Growth Assay

Upon the 50th passage, cells were cultured in 12 well plates, with 5 ×10^3^ cells per well, and maintained in DMEM supplemented with 10% FBS in a 5% carbon dioxide (CO2) humidified atmosphere at 37°C for 10 days. At 24-h intervals, three replicative wells were trypsinized and the cells were counted with a coulter counter. Once the cell growth curve was plotted, cell growth doubling time was calculated from its exponential growth phase.

### Real-Time PCR

Total RNA was extracted using TRIzol (Invitrogen) following the manufacturer's protocol. In brief, 0.8 μg of total RNA isolated from different samples was reverse transcribed using M-MLV reverse transcriptase (Promega) with an oligo(dT) 18 primer. Real-time PCR was performed using an UltraSYBR Mixture (Beijing CoWin Biotech, Beijing, China) and a ViiA 7 real-time PCR system (Applied Biosystems). GAPDH was used as a reference gene to analyze the dog's gene quantitatively. The primers used for HER2, ERα. PR,GAPDH (20), CDH1 (21), TGF-β (22), GATA3 (23), HIF1α (24), and β-actin (25) have all been previously reported. Specific primer sequences used for qRT–PCR assays are listed in Table 1.

**Table 1:** Primer sequences used for qRT-PCR.

**Gene**	**Forward primer**	**Reverse primer**
HER2	GGTCTTGATTCAGCGGAGCC	GGGCGTCTTACAAGCTGGA
ERα	TGGAGATCTTTGACATGTTGCTGGCTACG	GCTCCATGCCTTTGTTACTCATGTGCCTGA
PR	ACCTCCAGTTCTTTGCTGACGAGTC	GATCTCCATCCTAGTCCAAACACCA
CDH1	AAATCACATCCTACACCGCC	ATTAACCTCCAGCCAACCG
TGF-β	TAGTACACGATGGGCAGTGG	TGGACACGCAGTACAGCAA
GATA3	TACGTCCCCGAATACAGCTC	ACTCCCTGCCTTCTGTGCT
HIF1α	GTACTTCACTGCACAGGCCA	ACAAATCAGCACCAAGCACG
GAPDH	ATGGTGAAGGTCGGAGTCAA	ATCACCCCATTTGATGTTGG
β-actin	GCA TCC TGA CCCTCA AGT ACC	AGC TCG TTG TAG AAG GTGTGG

### Western Blot

Cells were either directly lysed in 2X sodium dodecyl sulfate (SDS) sample buffer or broken down ultrasonically, and then incubated at 100°C for 10 min. Protein samples were separated on a 12% SDS-PAGE gel and transferred to polyvinylidene fluoride (PVDF) membranes by electrophoresis. Membranes were blocked for 1 h at room temperature using 5% skim milk, followed by overnight incubation with primary antibody HIF1α (1:10,000), and incubated for 45 min at room temperature by Rabbit secondary antibodies (1:10,000). Reactive bands were developed and visualized with ImmobilonTM Western Chemiluminescent HRP Substrate (Millipore).

### Immunofluorescence

Immunofluorescence assay was performed to detect the expression of the HIF1α, vimentin in the B-CMT cell line, with MDCK cell line being used as a negative control. Cells culturing in a 48 well plate with glass coverslips were fixed in 4% paraformaldehyde for 30 min. Cells were then permeabilized for 10 min at 4°C and blocked for 30 min at room temperature (RT) using 1% bovine serum albumin (BSA), in succession. Cells were then exposed to primary antibodies and incubated for 45 min at RT, and subsequently washed and incubated with secondary FITC–anti-rabbit/mice IgG antibody for 45 min at RT. Finally, the cells were washed again and stained with DAPI for 3 min, and were observed through fluorescence microscopy.

### Generation of a B-CMT Cell Line That Exogenously Expresses EGFP

Plasmid including psin-EGFP, pspax2 and pmd2.g were preserved in our laboratory. HEK293T cells were seeded in 100-cm^2^ culture dishes and were then transfected with plasmids (10 μg of psin-EGFP, 10 μg of pspax2 plus 5 μg of pmd2.g). After 36–48 h of incubation, cell culture supernatant (virus) was harvested through filtration concentration technique and preserved at −80°C.

B-CMT cells were seeded in 24-well plates for 16 h and infected with or without virus plus a final concentration 8 μg/ml polybrene. Cells were infected again after 12 h of incubation. Finally, cells were screened by puromycin until uninfected cells were killed completely.

B-CMT cells stably expressing EGFP (EGFP-B-CMT) were generated by clonal experiments. Briefly, cell suspensions were serially diluted and seeded into 96-well plates. Wells containing individual cell were confirmed, and then single colonies were sub-cultured from each well into a 12-well plate and bigger petri dishes, sequentially.

### Tumorigenicity Assay

To estimate *in vivo* tumorigenic potential, a tumorigenicity assay was performed by subcutaneously injecting EGFP-B-CMT into the left mammary fat pad of 4-week-old nude mice (2 ×10^6^ cells per mouse, inoculating 3 mice). The mice were observed weekly for the growth of tumors. Mice were euthanized with sodium pentobarbital after 30 days post injection, and tumors and various tissues (cardiac, liver, spleen, lung, kidney, muscle) were collected at necropsy and fixed in 4% paraformaldehyde for histological and immunohistochemical examination.

### Immunohistochemistry

After fixing and embedding, canine tissue specimens were cut into 3 μm slices and fixed onto glass slides. Each specimen was deparaffinized for 1 h and underwent heat-induced antigen retrieval with high pressure-heated method in sodium citrate buffer (pH 6.3) for 5 min. The slides were covered with 3% H_2_O_2_ to eliminate *in-situ* tissue endogenous peroxidase activity after natural cooling, followed by incubation with 5% non-immunized goat serum at RT for 30 min. Sections were then incubated with primary antibodies for EGFP (1:100, sc-9996) overnight at 4°C and secondary antibody (goat anti-mouse IgG, pv-9002) for 45 min at RT to detect target proteins. Signal was visualized with 3, 3'-diaminobenzidine (DAB) and halted by washing in distilled water. Finally, the sections were re-dyed with hematoxylin, dehydrated, transparent and mounted. Specimens were also derived from mice inoculation with PBS as negative controls.

### MTT Assay

To verify the inhibitory effect of 5-Fluorouracil, Doxorubicin, Carboplatin, Rapamycin, and Imatinib on B-CMT cells, the MTT assay was performed. B-CMT cells were seeded into 96-well cell culture plates and incubated overnight at 37°C. Different concentrations of drugs were mixed with cells, respectively, on the following day and incubated for 48 h. A 20 μL volume of MTT (5 mg/mL) was added to each well and incubated again for 4 h. After discarding the supernatant, an aliquot of 150 μL of dimethylsulfoxide (DMSO) was added into each well and the plate was shaked to fully dissolve cristals. The absorbance at 490 nm (OD490) were measured in a microplate reader. The cell survival rate (%) was calculated by (OD_sample_/OD_control_) ×100.

### Cell Cycle Analysis

The cell cycle distribution of chemotherapeutic-treated cells (MEFs, B-CMT, and CMT7364) was detected by flow cytometry. The cells were mixed with different concentrations of chemotherapeutics and incubated for 24 h. Subsequently, cells were harvested and fixed for 1 h in 70% ethanol at room temperature. Then, the cells were centrifuged at 1,600 rpm for 5 min to remove the ethanol and washed with PBS, following by centrifugation again. The cells were stained with propidium iodide (PI) to generate PI-DNA complexes to detect cell cycle distribution (26).

## Results

### Histopathological Examination Revealed Mammary Mixed Adenocarcinoma

**Figure 1:**
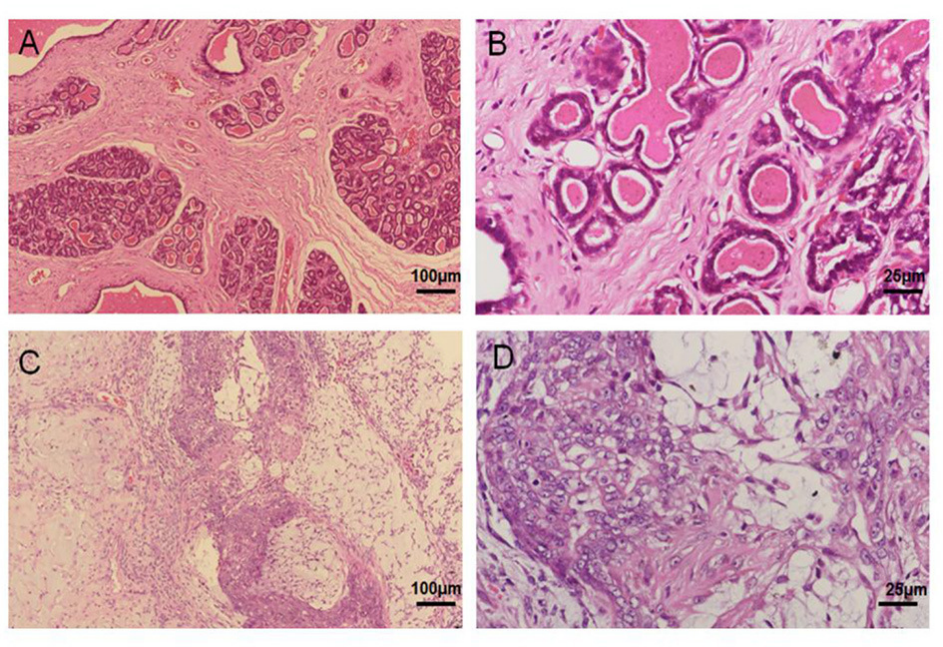
Primary canine mammary tumor. **(A,B)** Normal canine mammary tissue. **(C,D)** Canine mammary tumor tissue in paraffin sections showed a marked difference including numerous epithelial hyperplasia, cellular pleomorphism, partial calcification and poly-nuclear phenomenon.

Mammary cancer tissue was derived from a 13-year-old female sterilized Pomeranian dog. The histopathological reports notarized the diagnosis of mammary mixed adenocarcinoma via H&E staining analysis; however, there was no metastasis in local lymph nodes and distal organs via CT examination. As compared to normal mammary tissue structure (Figures 1A,B), severe epithelial hyperplasia, cellular pleomorphism, and partial calcification were observed in mammary gland cancer tissue. In addition, the neoplasm was enveloped with fibrous capsule, and the normal mammary gland structure had been replaced by a large number of proliferative glandular epithelial cells with vigorous division, along with myoepithelial cells. The cells were characterized by irregularly sized lobules, multi-layered arrangement, and tenuous fibrous vascular matrix invasion of tumor cells, and island-like distribution of glandular epithelial cell (Figures 1C,D). Based on the histological examination, the tumor was diagnosed as a canine mammary mixed adenocarcinoma.

### Microscopic Morphology of B-CMT and Growth Assay

**Figure 2:**
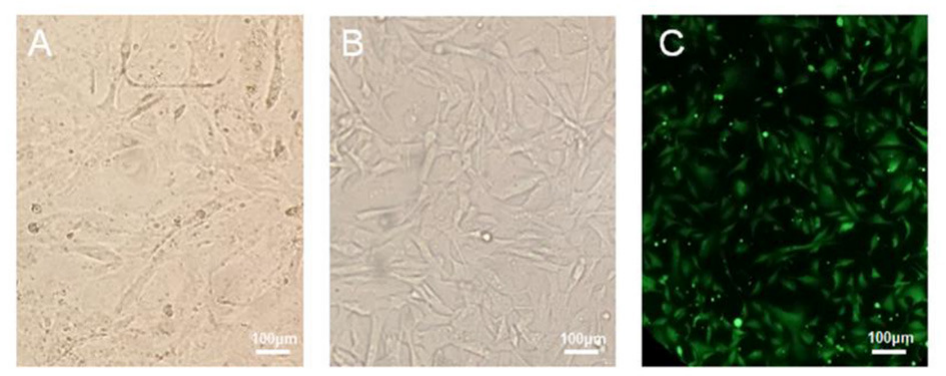
Morphology of canine mammary cancer B-CMT cell line. **(A,B)** B-CMT cell line at passage 6 and 52, respectively. **(C)** B-CMT cell line exogenously expresses EGFP by lentivirus packaging. Light microscopy showed the cells were lean to polygon-shaped.

The canine mammary cancer cell line was maintained in culture medium containing 10~20% FBS, for a minimum of 50 passages, over a 1-year period. The successfully established cell line was named as B-CMT. Cell morphology of the 6th and 52nd passages are presented in Figures 2A,B, respectively. The initial few passages of B-CMT cells show pleomorphism, with round or oval cells, polygonal cells and a few spindle cells being observable. With the increase of cell passage number, the morphology of cancer cells tended to be consistent. Majority of these cells were polygonal (Figure 2B). Moreover, B-CMT cells stably expressing EGFP (EGFP-B-CMT) were successfully generated by clonal experiments, which allowed the cancer cells to be further purified (Figure 2C). EGFP-B-CMT is very important for the detection of cancer cell *in vivo*.

**Figure 3:**
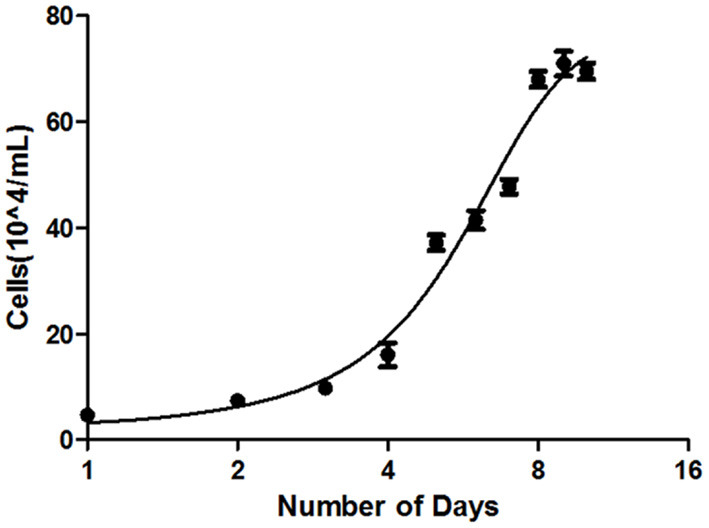
Population doubling time of B-CMT was calculated to be 33.6 h.

Growth assay and population doubling time of the B-CMT cell line were determined as previously describes in the present study materials and methods. The B-CMT cell line exhibited an “S-shaped” growth curve as shown in Figure 3, indicating that the proliferation experienced distinct delay phase, logarithmic phase and plateau phase. The population doubling time (PDT) of B-CMT cells was 33.6 h.

### B-CMT Cell Line Characteristics

In order to determine whether this cell line is a triple negative mammary gland cancer cell, we used RT-PCR to detect the mRNA expression levels of canine estrogen receptor alpha (ERα) and progesterone receptors (PR), as well as HER2/c-erbB-2/Neu (HER2). In addition, because E-cadherin (CDH1) expression tends to be significantly reduced in cancer cells, we also included detection of its mRNA expression levels. CMT cell lines (CMT7364 and B-CMT) and MDCK cell cultures were used as models of mammary cancer cells and normal cell control, respectively.

**Figure 4:**
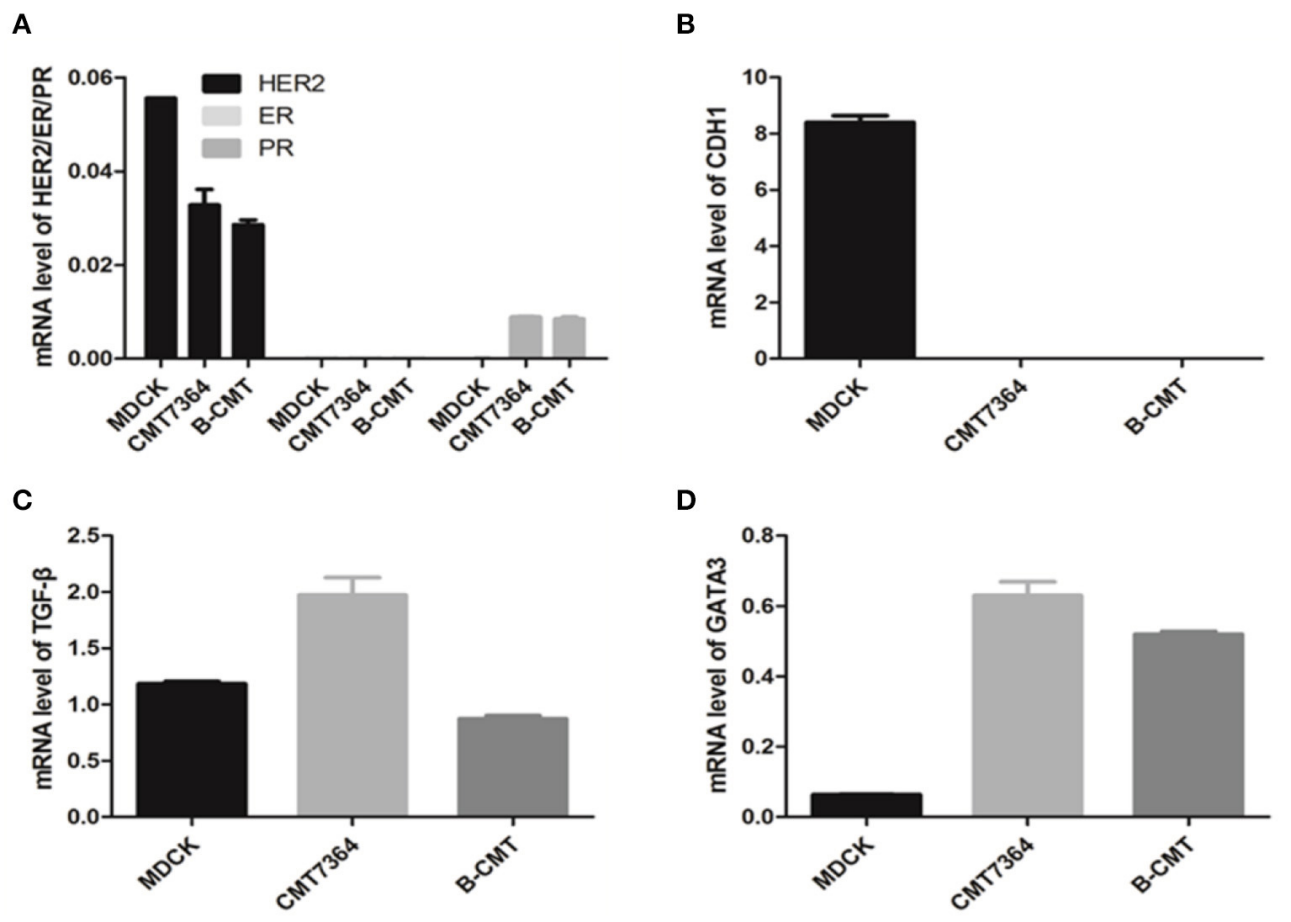
Expression of canine ERα, PR, HER2, CDH1, TGF-β, and GATA3 in canine mammary tumor B-CMT Cells. In order to detect expression levels for each gene in total cell RNA isolated from B-CMT, quantitative reverse transcriptase polymerase chain reaction (qRT-PCR) assays were used to discreetly amplify mRNA of each relating canine gene. Among cells used, MDCK and CMT7364 were used as control cells. **(A)** The expression of canine ERα, PR, and HER2; **(B)** The expression of canine CDH1; **(C)** The expression of canine TGF-β. **(D)** The expression of canineGATA3. GAPDH or β-actin were used as a reference gene to analyze the dog gene quantitatively in this assay. All relative levels of mRNA was calculated by 2^−(*Cqsample*−*CqGAPDH*−5)^.

Gene expression was normalized to an internal reference control gene, GAPDH. Relative gene expression levels from qPCR data were calculated by using the ΔΔCq calculation method. qRT-PCR analysis of ERα, PR, and HER2 gene revealed trace level expression in MDCK, CMT7364, B-CMT cell lines (Figure 4A). Compared with MDCK cells, CDH1 expression significantly was decreased, even absent in B-CMT cells (Figure 4B); TGF-β expression was increased on CMT7364 while remain at similar level on B-CMT (Figure 4C). However, GATA3 expression was dramatically increased in both CMT7364 and B-CMT (Figure 4D). The result demonstrated B-CMT cell line was a triple-negative canine mammary cancer cell line.

**Figure 5:**
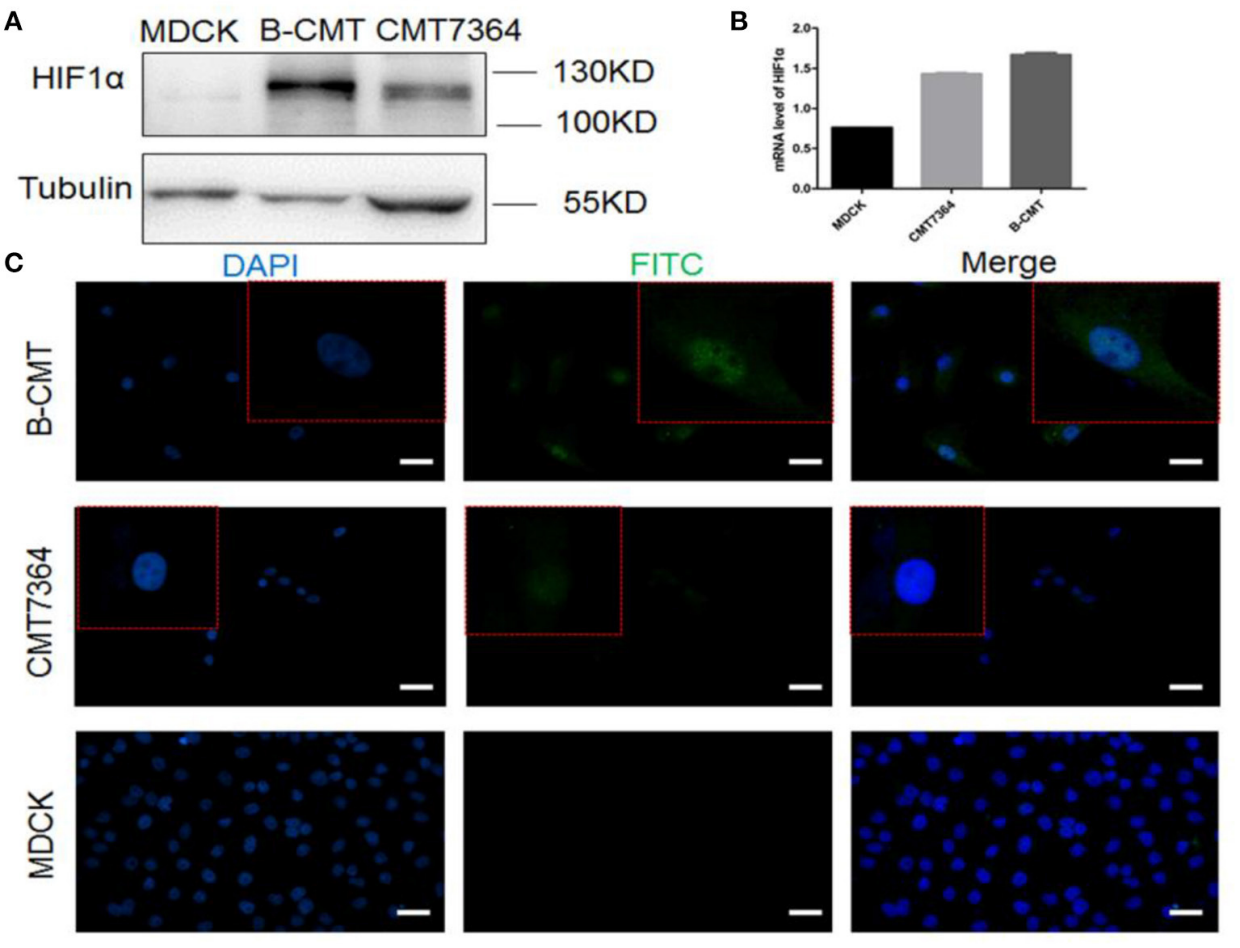
The expression of HIF1α in B-CMT cell line. **(A,B)** HIF1-α is highly expressed in B-CMT cell line compared to MDCK and CMT7364 cell lines by western blot and qRT-PCR; **(C)** HIF1-α is located in cell nucleus with B-CMT cell line by immunofluorescence, and MDCK and CMT7364 cell lines were used as control cells.

Hypoxia-inducible factor 1 (HIF-1) is a transcription factor that regulates the expression of downstream genes which play important roles in many critical aspects of cancer progression, including angiogenesis, metabolism, stem cell renewal, immune avoidance and therapeutic resistance (27). The expression of HIF1-α in B-CMT was detected by Western blot and qRT-PCR (Figures 5A,B). Compared with MDCK cells, HIF1-α is highly expressed in CMT cell lines, especially B-CMT cells. Additionally, HIF1-α is mainly concentrated in the nucleus, which is where many researchers believe it can regulate gene transcription in many aspects of cancer (Figure 5C).

### Tumorigenic Analysis

**Figure 6:**
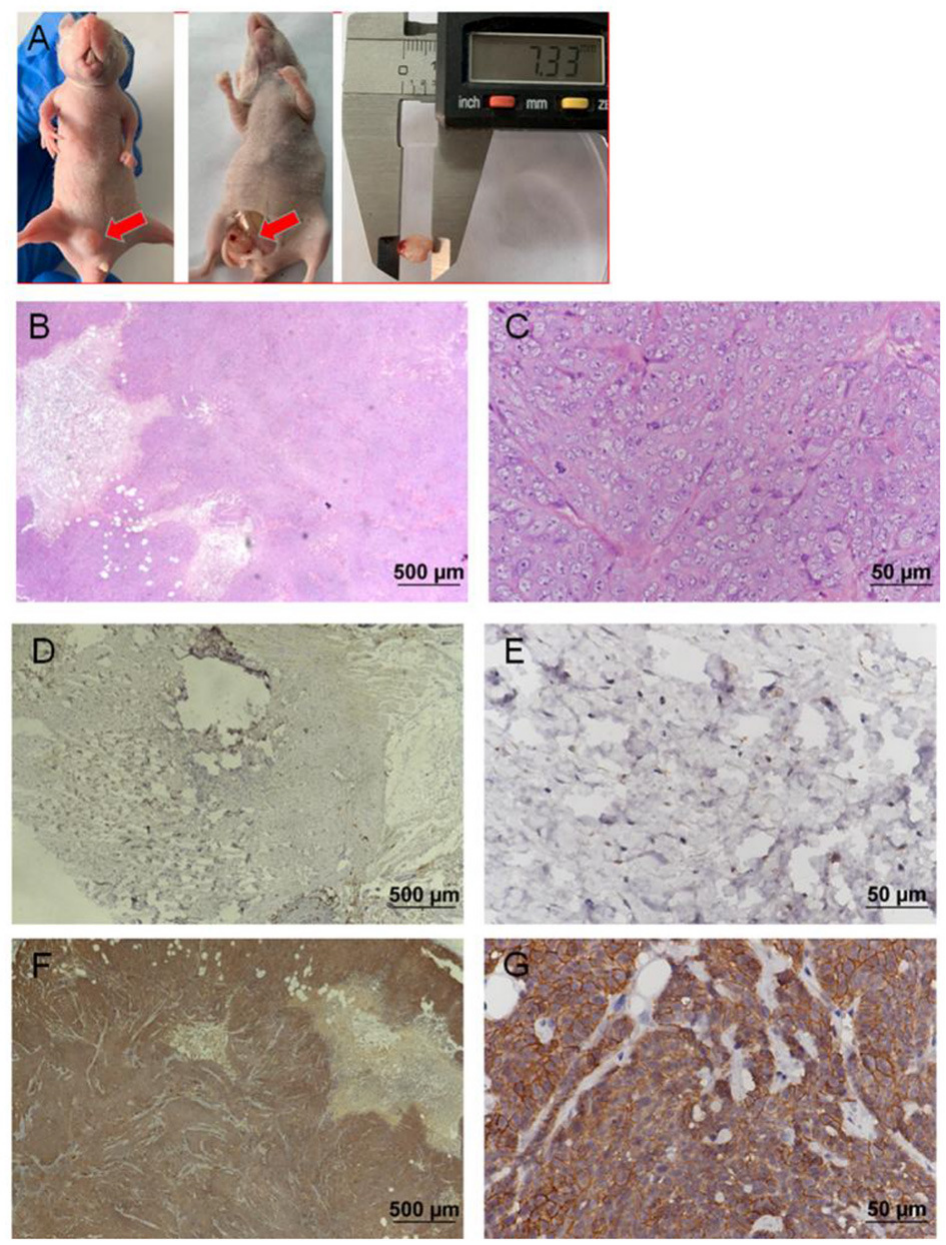
Nude mouse tumorigenicity assay. 2 ×10^6^ B-CMT cells were subcutaneously injected into the mammary fat pad of immunodefcient nude mice. Red arrows indicated the tumors in the nude mice. **(A)** One nude mice 30 days post-injection of B-CMT cells, where tumors appeared on mammary fat pad; **(B,C)** The pathological findings of mammary tumor tissue from xenografted mice was performed by H&E. **(D,E)** CMT7364 cells (negatively expressing EGFP), a negative control cell line, were injected subcutaneously into mice, and xenografted mice was euthanatized after inoculation 30 days as well as tumor paraffin sections was used to detect the expression of EGFP by immunohistochemistry (IHC). **(F,G)** B-CMT cells (stably expressing EGFP) were injected subcutaneously into mice, and xenografted mice was euthanatized after inoculation 30 days as well as tumor paraffin sections was used to detect the expression of EGFP by immunohistochemistry (IHC).

EGFP-B-CMT canine mammary cancer cells developed tumors after 14 days post inoculation (Two of the three nude mice developed tumors). The nude mice developed a touchable soft tissue mass of 7.33 mm of diameter that was detected at the injection site 30 days after injection (Figure 6A). Histological examination indicated a highly infiltrating, poorly demarcated solid tumor that damaged the normal structure of the mammary gland (Figures 6B,C), and the morphological characteristics of the nude mouse transplanted tumor was similar with that of the primary tumor (Figures 1C,D). However, there was no obvious metastasis of canine mammary cancer in main organs such as heart, liver, spleen, lungs, kidney and muscle within 30 days (Supplementary Figures 1A–L). In addition, the nude mice inoculated with PBS did not develop any tumor (No data shown).

To further determine whether a soft tissue mass is a tumor that develops from B-CMT mammary gland cancer cells, we examined the expression of GFP in soft tissue mass by IHC. IHC examination revealed that CMT7364 cell lines (do not express EGFP) were negative for EGFP (Figures 6D,E), but B-CMT cell lines (stably expressing EGFP) were strongly positive for EGFP in the cytoplasm and membrane (Figures 6F,G). The result indicated that B-CMT cell lines are tumorigenic in immune-deficient mice, but might not have the potential to metastasize.

### Evaluation of Inhibitory Effects of Drugs on the Growth of Cells

**Figure 7:**
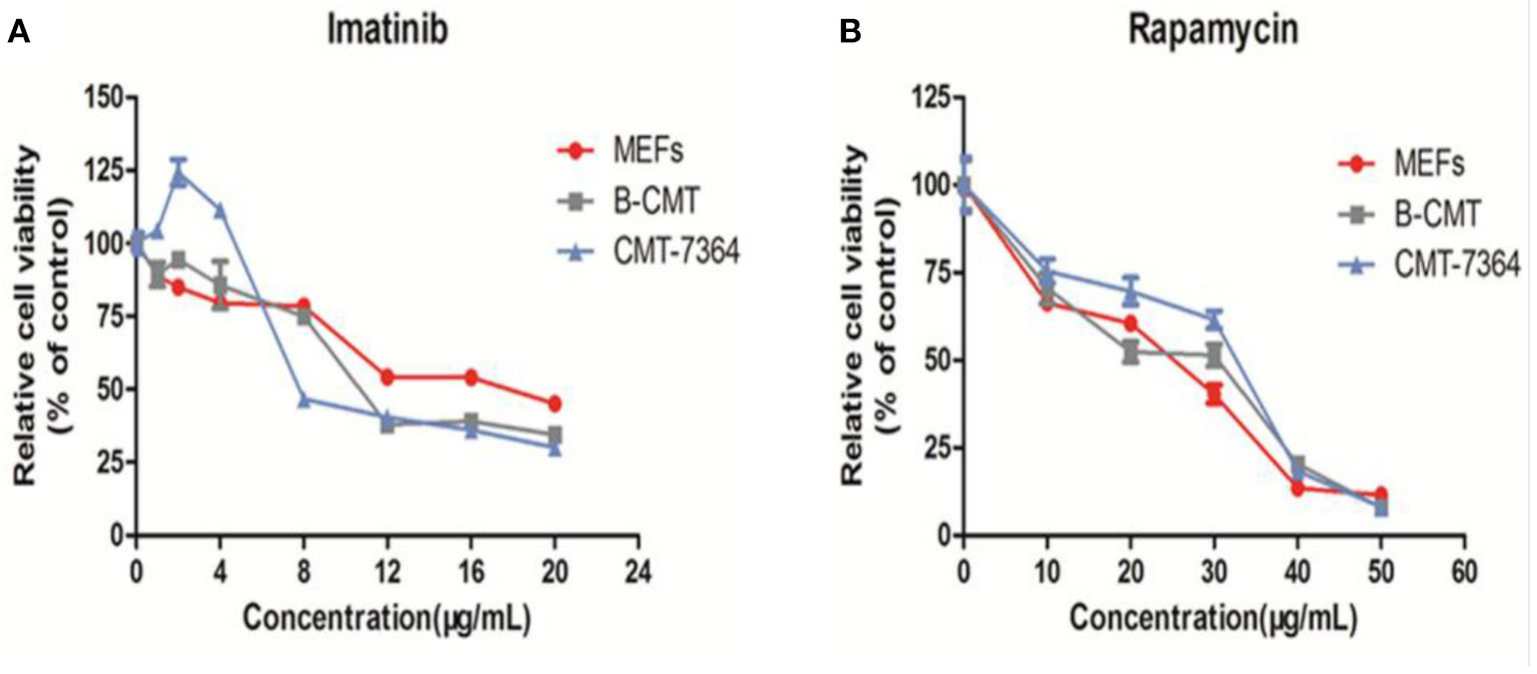
Anti-proliferative activity *in vitro* of chemotherapy drugs. **(A)** Anti-proliferative activity *in vitro* of rapamycin against cells at 10, 20, 30, 40, and 50 μg/mL after 48 h of treatment; **(B)** Anti-proliferative activity *in vitro* of imatinib against cells at 1, 2, 4, 8, 12, 16, and 20 μg/mL after 48 h of treatment. MEFs and CMT7364 cell lines were served as control cells, and vehicle DMSO were used as control.

The inhibition of cell growth by five drugs including 5-Fluorouracil, doxorubicin, carboplatin, rapamycin and imatinib was detected on MEFs, CMT7364 and B-CMT cell lines. Dose-dependent curves were drawn based on the relative cell viability at multifarious concentrations of drug. B-CMT cells were not sensitive to the first three drugs, especially being extremely insensitive to doxorubicin (Supplementary Figure 2). Rapamycin and imatinib have rarely been reported in the treatment of canine mammary gland tumors, however they are also commonly used as adjuvant drugs in cancer treatment and have growth inhibition effect on many cancer cell lines. Therefore, we wanted to determine whether either of these drugs had an inhibitory effect on the growth of B-CMT cell lines with CMT7364 and MEF cells serving as controls. Growth of B-CMT cell line was inhibited by treating with both drugs (Figures 7A,B). Moreover, there were significant differences in relative survival rate between B-CMT and CMT7364 cell lines at low drug concentration of imatinib, but relative survival rate of B-CMT cell line was contrary to that of MEFs cell line at high concentration of imatinib (*P* <0.01) (Figure 7B).

### Chemotherapeutic Induced Cell Cycle Arrest

**Figure 8:**
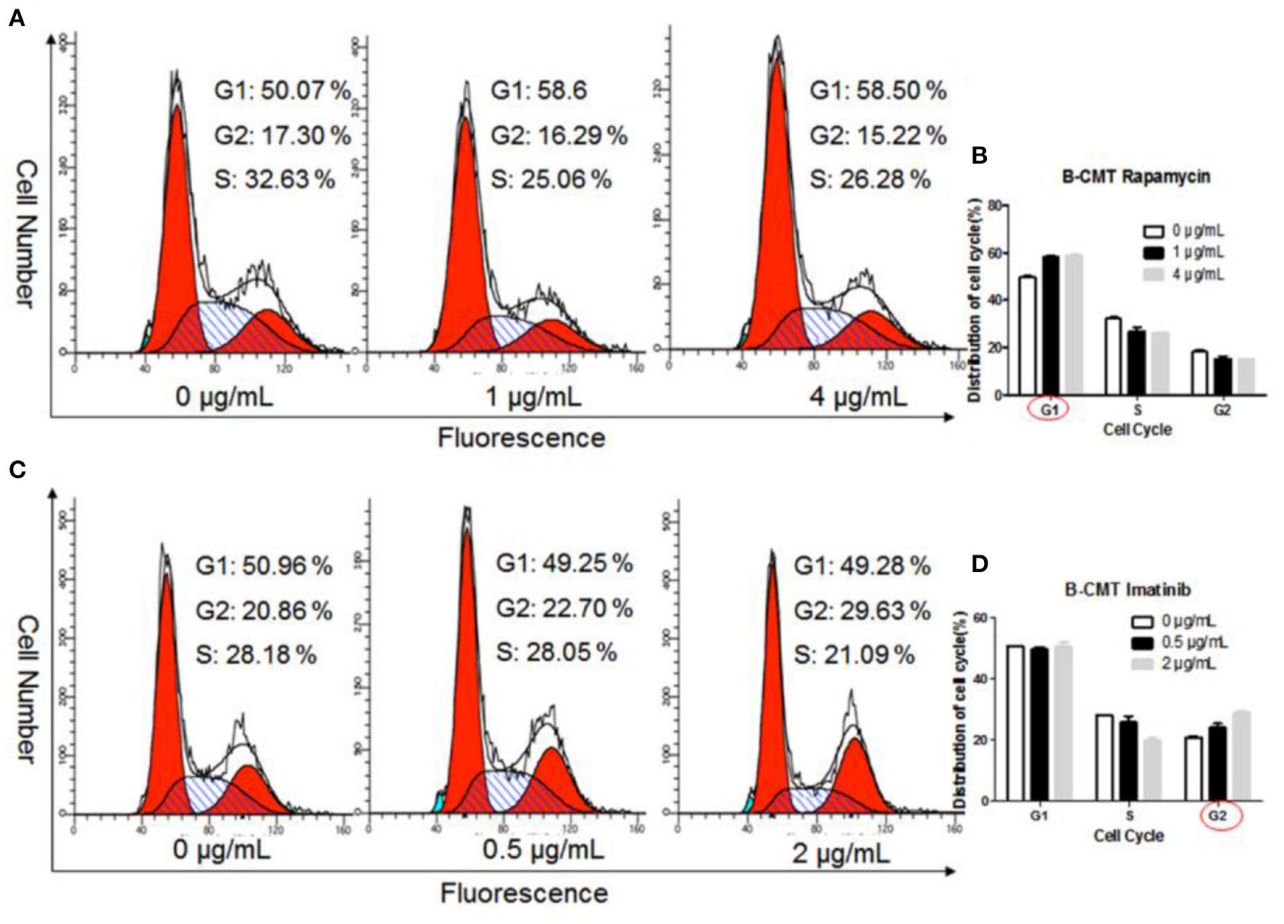
Chemotherapeutics induce cell cycle arrest. **(A)** Flow cytometry was used to examine the cell cycle distribution of Rapamycin-treated B-CMT cells; **(B)** Statistical analysis of the cell cycle distribution of B-CMT cells treated by Rapamycin; **(C)** Flow cytometry was used to examine the cell cycle distribution of Imatinib-treated B-CMT cells; **(D)** Statistical analysis of the cell cycle distribution of B-CMT cells treated by Imatinib. Red circles indicated cell cycle arrest in this cycle. MEFs and CMT7364 cell lines were used as control cells.

To further explore the inhibitory effect of chemotherapeutics on the proliferation of B-CMT cells, we determined the cell cycle distribution of B-CMT cells after 24 h of treatment with chemotherapeutics. Flow cytometry analysis indicated that, comparing to control group, the proportion of B-CMT cells at G1 and G2 phase was significantly increased after treatment with rapamycin and imatinib (Figures 8A–D), which in consistent with results of both MEFs and CMT7364 cells (Supplementary Figures 3A–H).

## Discussion

Spontaneously occurring canine mammary tumors share some common molecular characteristics and biological behavior with that of human breast cancer (28). This would lead to the strong consideration of a canine mammary tumor model being a more appropriate or ideal model for human breast cancer (29) as opposed to a murine model, for example, which is induced and does not spontaneously occur.

Canine mammary gland cancer cell lines are of great significance for us to further understand the development of canine mammary gland cancer, which would in turn provide a novel cell model for human breast cancer research. In this study, originally isolated from a primary canine mammary gland cancer, a novel canine mammary cancer cell line was successfully established for a minimum of 50 passages over the period of 1 year, and was designated B-CMT. A B-CMT single cell can replicate and form a community of cells to provide the basis for further research. Although, there are already many pre-existing mammary gland cancer cell lines in humans and dogs (30–32), each line has its own unique properties, which is particularly important to enrich the variety and quantity of cell lines, as well as providing the foundation for the development of translational medicine. Overall, we established a novel canine mammary cancer cell line, designated B-CMT which exhibits rapid proliferation by adherent growth with a doubling time of 33.6 h.

In addition to standard pathological analysis for the diagnosis of mammary gland cancer, gene expression profiles of mammary gland tumor biopsies can be determined using microarray analysis (20). Quantitative reverse transcriptase polymerase chain reaction (qRT-PCR), a simple and convenient, as well as a quantitative assay, can detect canine mammary cancer mRNA transcripts to determine/confirm canine mammary gland cancer subtypes that can be subsequently targeted for precise effective therapy. In this study, qRT-PCR assays were performed to successfully validate five genes (ERα, PR, HER2, E-Cadherin, and HIF1-α) that can be used to aid in determining mammary cancer subtype and biological characteristics.

Estrogen receptor (ERα or ER1), progesterone receptor (PR) and HER2 receptor tyrosine kinase are key indicators for determining the type of mammary gland cancer including luminal A, luminal B, HER2-positive, and basal/triple negative phenotypes (33–35). These gene expression profiles are of great significance for early diagnosis of diseases and clinical outcome as well as treatment response (36). qRT-PCR results showed that the ERα, PR and HER2 genes expressed only trace levels among MDCK, CMT7364, and B-CMT cell lines. MDCK and CMT7364 cell lines served as control cells. The CMT7364 cell line has been confirmed to be a triple negative mammary gland cancer (TNBC) (37); therefore, we determined that B-CMT is also a triple negative mammary gland cancer cell line.

E-cadherin are glycoproteins mediating cell-cell adhesion and play an important role in development, invasion, and metastasis of tumors. The expression of E-cadherin is down-regulated when the intercellular connection is destroyed, which will lead to the invasion and metastasis of tumor cells, suggesting that E-cadherin expression is related to the differentiation, invasion and metastasis of malignant tumors (38). Additionally, E-cadherin has been reported to play a role in the occurrence and development of canine mammary gland tumors (39). Compared with the MDCK cell line, qRT-PCR results revealed that B-CMT and CMT7364 lacked E-cadherin expression, which has already been demonstrated in CMT7364 cells. There was no difference in the mRNA expression of TGF-β between MDCK and B-CMT cell lines; however, the CMT7364 cell line had an increased TGF-β expression that was beneficial to the metastasis of the tumor, which was consistent with previous reports (37). GATA3 is a zinc finger transcription factor that is critical for the differentiation of mammary luminal epithelium (40). Shaoxian et al. (41) who reported that GATA3 was expressed in 82.83% of invasive breast cancers. We also demonstrated that GATA3 was highly expressed in canine mammary gland tumors in our study.

HIF1-α, a transcription factor, promotes the development of tumors. HIF1α has a variety of biological functions, which are closely related to metabolism (42), angiogenesis (43), cell proliferation (44), differentiation and drug resistance (45). In our study, we detected high level of HIF1-α expression at the protein level through western blot, immunofluorescence (IF) and qRT-PCR. IF analysis showed that HIF1-α was mainly localized in the cell nucleus in the B-CMT cell line. Therefore, stable HIF1α-expressing B-CMT cell line is a valuable cell model for HIF studies.

B-CMT cells were successfully inoculated into nude mice and resulted in palpable mass. Two out of three mice successfully developed tumors when inoculated, therefore, the cells were relatively prone to tumorigenesis. The histological examination of the mass in transplant tumor nude mice were similar with those of the canine primary tumor lesions. Additionally, histological findings indicated that this canine primary tumor did not generate distal metastasis to heart, liver, spleen, lungs, kidney and muscle within 30 days, but which do not exclude the possibility of inoculated tumor to metastasize long time later. We successfully overexpressed EGFP in B-CMT and established a canine mammary gland cancer xenograft tumor model with EGFP-B-CMT cell line. Furthermore, the xenograft tumor was derived from B-CMT cell line by detecting the expression of EGFP using IHC.

The results of drug screening *in vitro* indicated a variable sensitivity between the various drugs and the cell lines. In the drug screening section, MDCK cells are used as the control of normal cells because it grows too fast, so there is little significance to use it as the control of canine mammary gland cancer cells. Unfortunately, it is difficult to obtain canine normal mammary gland cancer cells. Therefore, MEFs was successfully established in our laboratory that was considered as normal cells, and the cell growth rate is basically similar that of canine mammary gland cancer cells.

*In vitro* cytotoxicity test results demonstrated that the five drugs had different effects on the three cells at variable drug concentrations. First, we found that rapamycin and imatinib could effectively inhibit cell proliferation of B-CMT. Rapamycin and imatinib have rarely been reported in the treatment of canine mammary gland tumors, however they are commonly used as adjuvant drugs in cancer treatment and have demonstrated a growth inhibitory effect on many cancer cell lines. Second, B-CMT was demonstrated a high level of drug resistance to doxorubicin. This phenomenon may be related to the high expression levels of HIF1α in the B-CMT cell line which may be acting via 2 possible pathways. Drug resistance may be induced by HIF-1α-mediated P-gp expression which has been investigated in a variety of tumor cells, including mammary gland carcinoma and colon cancer cells (46, 47). HIF-1α inhibited doxorubicin-mediated apoptosis has also been reported (48).

Finally, in order to determine whether sensitive drugs including rapamycin and imatinib could inhibit cell growth by blocking cell cycle, we examined cell cycle distribution using flow cytometry. The results have demonstrated that it was a variable cell cycle arrest between both drugs, and across the cell lines. Previous studies have reported that cannabidiol could arrest SGC-7901 cells at the G0–G1 phase (49). Therefore, Rapamycin and imatinib may be candidates for treatment of this type of mammary gland cancer.

## Conclusion

The use of spontaneous mammary gland cancer in the dog as a translational model for human mammary cancer provides an opportunity to further understand this category of malignancy, and to explore better treatment strategies. In this study, a novel canine mammary cancer cell line, B-CMT, was successfully established and characterized on a preliminary basis and explored for sensitivity of five chemotherapeutic drugs to cells. Interestingly, studies have demonstrated that B-CMT is characterized by a high expression level of the hypoxia-inducing factor HIF1α, which may be significantly contributing to its resistance to doxorubicin. Rapamycin and imatinib had a significant effect on B-CMT cell cycle arrest. The cell line may have great research significance for future studies on tumor microenvironment, drug resistance and mammary gland cancer treatment. It is worth mentioning that established mammary gland tumor cell lines are widely used in cancer research. Furthermore, the ideal cell models are important research tools in bringing insights into the related mechanisms of mammary gland cancer, as well as in delivering improved therapies for the disease. Therefore, we believe that B-CMT is a valuable canine cell line and will have great impact on exploring cancer treatments between human as well as dogs.

## Data Availability Statement

The original contributions presented in the study are included in the article/Supplementary Material, further inquiries can be directed to the corresponding author/s.

## Ethics Statement

The animal study was reviewed and approved by All animal procedures and study design were approved by the animal ethics committee of China Agricultural University.

## Author Contributions

GL, RL, JT, and YK designed the whole study. RL and GL performed the experimental work and data analysis. HW, YS, and JZ taken part some of the experiments. RL wrote the manuscript. GL and JT revised the manuscript. The manuscript was read and approved by all authors.

## Conflict of Interest

The authors declare that the research was conducted in the absence of any commercial or financial relationships that could be construed as a potential conflict of interest.

## Abbreviations

PDT: population doubling time
HER-2: human epidermal growth factor receptor-2
ER: estrogen receptors
PR: progesterone receptors
HIF-1α: hypoxia inducible factor-1α
GATA3: endothelial transcription factor 3
IMC: canine inflammatory mammary cancer
IHC: Immunohistochemistry.
