# Supplementary material for: A Novel Canine Mammary Cancer Cell Line: Preliminary Identification and Utilization for Drug Screening Studies
Source: Front Vet Sci. 2021 May 27;8:665906. doi: 10.3389/fvets.2021.665906 (PMC8191460; doi:10.3389/fvets.2021.665906)
Supplement: Supplementary file 1 [file Data_Sheet_1.docx]

Supplementary Material

# Supplementary Figures

## Supplementary Figures


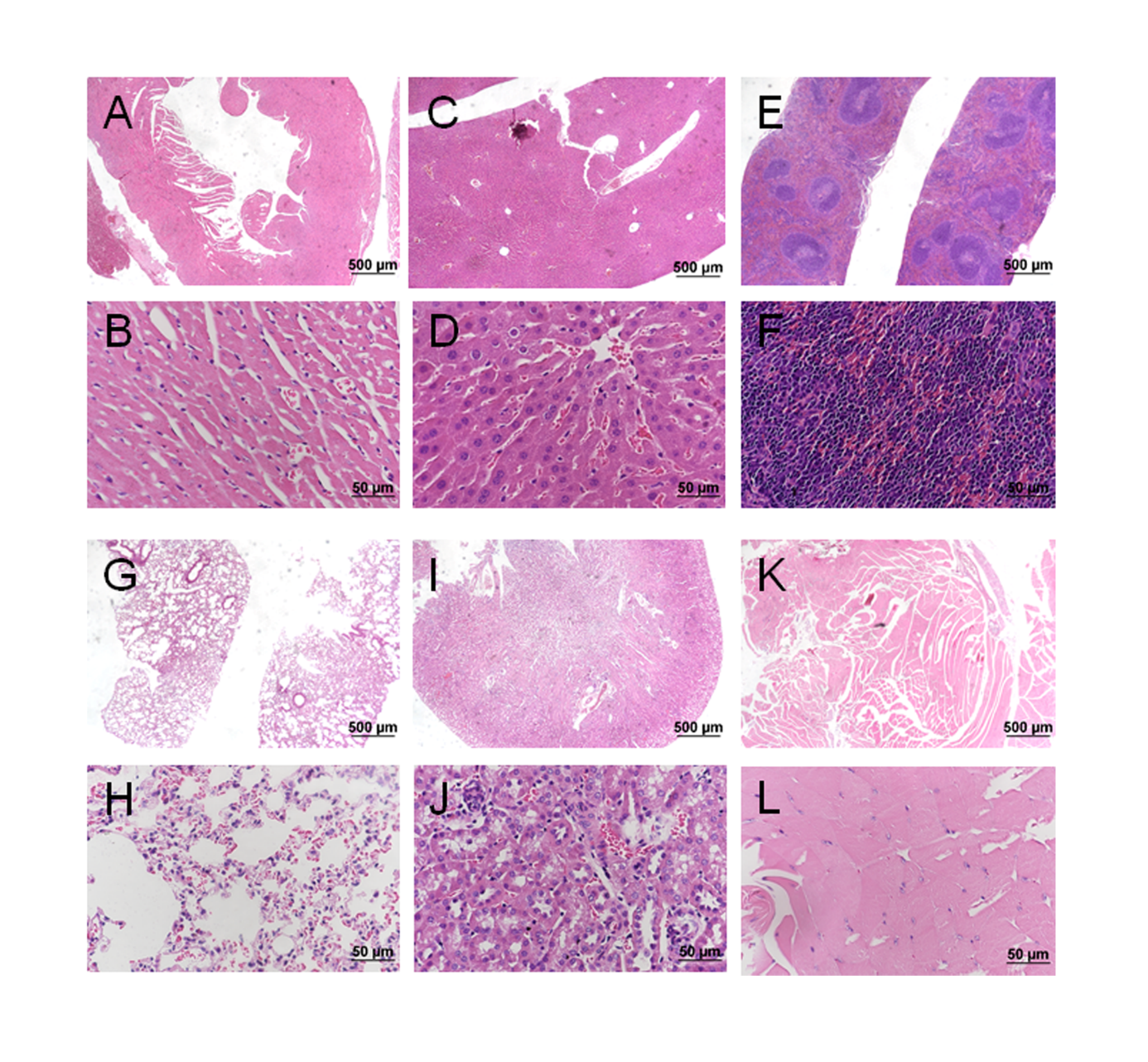


**Supplementary Figure 1.** **Nude mouse tumorigenicity assay**. Detecting metastasis of canine mammary cancer in mainly organs by H&E. (A-B) Heart; (C-D) Liver; (E-F) Spleen; (G-H) Lung; (I-J) Kidney; (K-L) Muscle.

**
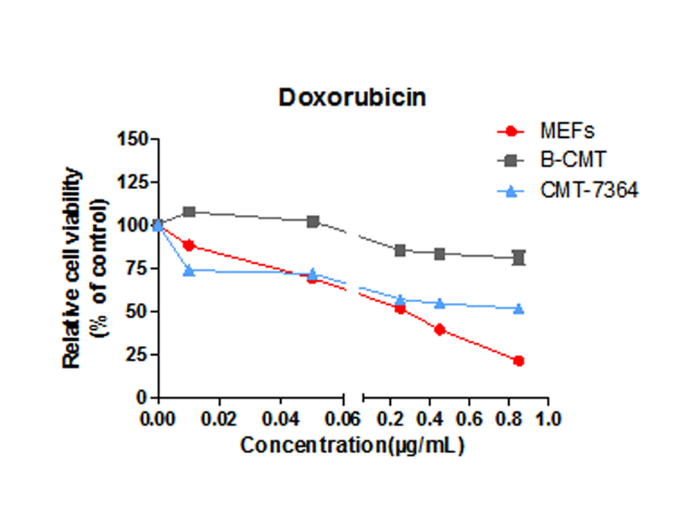
**

**Supplementary Figure 2. Anti-proliferative activity in vitro of Doxorubicin.** Anti-proliferative activity in vitro of Doxorubicin against cells at 0.01, 0.05, 0.25, 0.45, 0.85 μg/mL after 48 h of treatment.

**
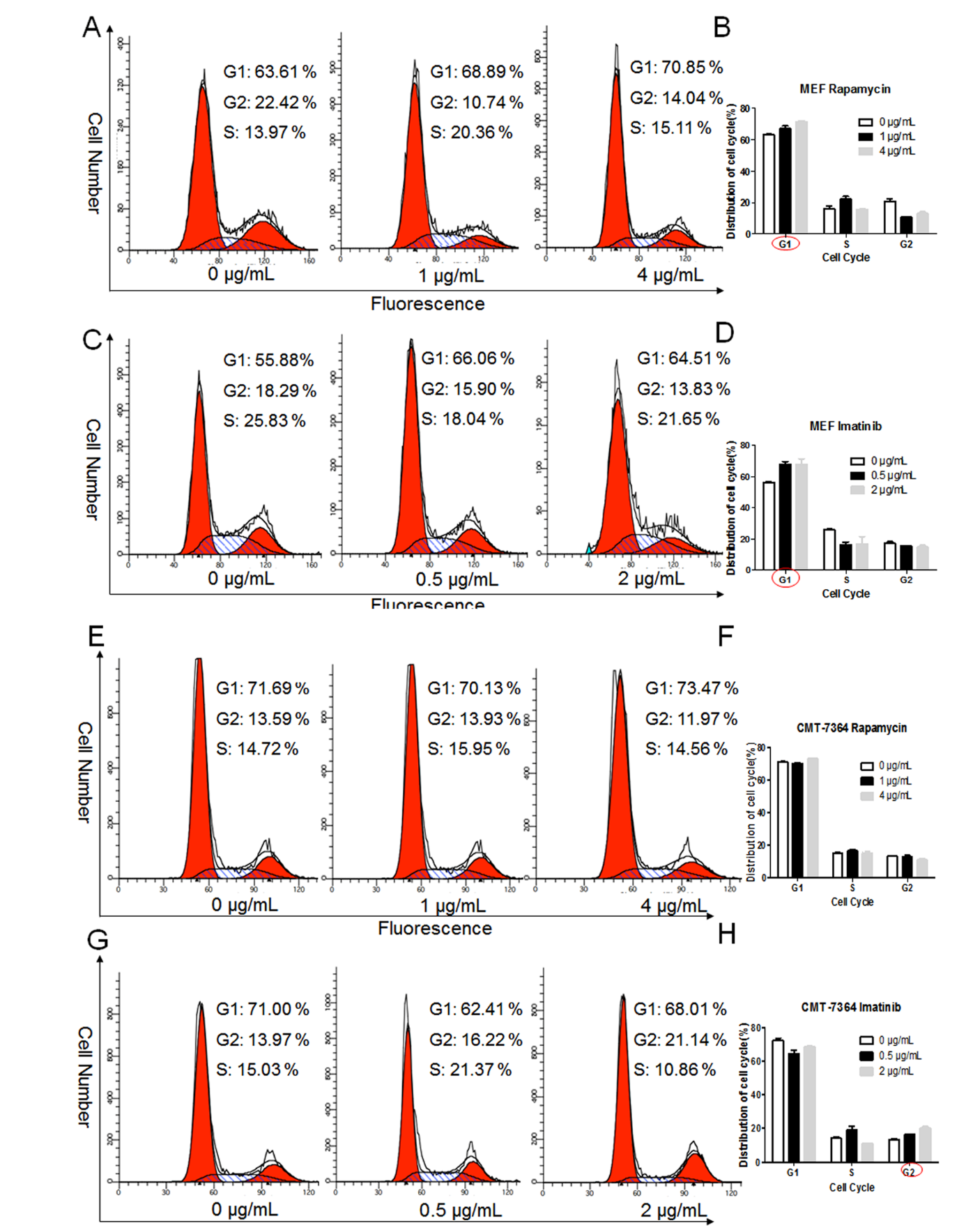
**

**Supplementary Figure 3. Chemotherapeutics Induce Cell Cycle Arrest**. (A) Flow cytometry was used to examine the cell cycle distribution of Rapamycin-treated MEFs cells; (B) Statistical analysis of the cell cycle distribution of MEFs cells treated by Rapamycin; (C) Flow cytometry was used to examine the cell cycle distribution of Imatinib-treated MEFs cells; (D) Statistical analysis of the cell cycle distribution of MEFs cells treated by Imatinib. (E) Flow cytometry was used to examine the cell cycle distribution of Rapamycin-treated CMT7364 cells; (F) Statistical analysis of the cell cycle distribution of CMT7364 cells treated by Rapamycin; (G) Flow cytometry was used to examine the cell cycle distribution of Imatinib-treated CMT7364 cells; (H) Statistical analysis of the cell cycle distribution of CMT7364 cells treated by Imatinib. Red circles indicated cell cycle arrest in this cycle.
